# Long-term remission of myopic choroidal neovascular membrane after treatment with ranibizumab: a case report

**DOI:** 10.1186/1752-1947-3-84

**Published:** 2009-10-28

**Authors:** Neruban Kumaran, Dawn A Sim, Adnan Tufail

**Affiliations:** 1 Department of Medical Retina, Moorfields Eye Hospital, London, UK

## Abstract

**Introduction:**

Myopia has become a big public health problem in certain parts of the world. Sight-threatening complications like choroidal neovascularisation membranes occur in up to 10% of pathological myopia, and natural history studies show a trend towards progressive visual loss. There are long-term financial and quality-of-life implications in this group of patients, and treatment strategies should aim for long-term preservation of vision.

**Case presentation:**

A 56-year-old Caucasian woman presented with a best-corrected visual acuity of 6/6-1 in her right eye and 6/24 in her left. Fundal examination revealed pathological myopia in both eyes and an elevated lesion associated with pre-retinal haemorrhage in the left macula. Ocular coherence tomography and fundus fluorescein angiogram confirmed a subfoveal classic choroidal neovascularisation membrane. The patient decided to proceed with intravitreal ranibizumab (0.5 mg) therapy. One month after treatment, best-corrected visual acuity improved to 6/12 in her left eye, with complete resolution subretinal fluid on ocular coherence tomography. After three months, best-corrected visual acuity further improved to 6/9, which was maintained up to 16 months post-treatment.

**Conclusion:**

We suggest intravitreal ranibizumab as an alternative treatment for long-term remission of myopic choroidal neovascular membrane. It also suggests that myopic choroidal neovascularisation membranes may require fewer treatments to achieve sustained remission. Furthermore, this could serve as a feasible long-term management option if used in conjunction with ocular coherence tomography.

## Introduction

In certain parts of the world, myopia has reached epidemic proportions and is now a major public health problem [1]. The prevalence of high and pathological myopia appears to be rising in Asia and other parts of the world. This has a large public health impact because of the associated increase in potentially blinding ocular complications. High myopia or myopia with increased risks of ocular morbidity can be defined as a spherical equivalent of at least -6 OD. The resulting ocular pathology is usually due to excessive elongation of the eyeball and associated with pathological changes in the fundus [2].

Myopia accompanied by degenerative changes in the sclera, choroid, retinal pigment epithelium and associated compromises in visual function have also been termed 'degenerative', 'malignant' and 'pathological' [3].

Many complications and associations have been noted with such 'pathological' myopia. Evidence from both clinic and population-based studies suggest that high and low myopia in European and Afro-Caribbean populations [4,5] may be associated with cataract (posterior subcapsular, nuclear and occasionally, cortical cataract), the leading cause of blindness in the world [6].

Myopic eyes are known to have longer axial lengths and vitreous chamber depths compared to emmetropic eyes. Eyes with longer axial lengths tend to have higher cup-disc ratios, increased optic nerve fibre layer defects and possibly greater deformity of the lamina cribrosa, leading to high susceptibility to glaucomatous optic disc changes [7]. Such elongation may lead to mechanical stretching and thinning of the choroid and retinal pigment epithelium and other vascular degenerative changes. These changes include choroidal neovascularisation, macular holes, chorioretinal atrophy, Fuchs' spots, lacquer cracks, lattice degeneration and retinal breaks. Here, we describe the presentation, follow-up and management of a myopic patient who presented with a choroidal neovascular membrane (CNVM), as a result of choroidal neovascularisation (CNV).

## Case presentation

A 56-year-old Caucasian woman with high-myopia (-6.00) presented with a one month history of sudden, painless distortion of vision in her left eye. She noted that reading had been more difficult for the last two weeks. Previous documented best corrected visual acuity (seven years ago) was 6/5 in the right eye and 6/6 in the left eye. Previous ocular history of note was macular change secondary to myopia, diagnosed by her optician eleven years previously. Both of her parents were myopic, but her medical history was otherwise unremarkable.

On examination, best-corrected visual acuities were 6/6-1 in the right and 6/36, improving to 6/24 with pinhole, in the left eye. The left eye was noticed to have an Adie (tonic) pupil. Both anterior segments were deep and quiet and the intraocular pressure was 16 mmHg in each eye.

**Figure 1:**
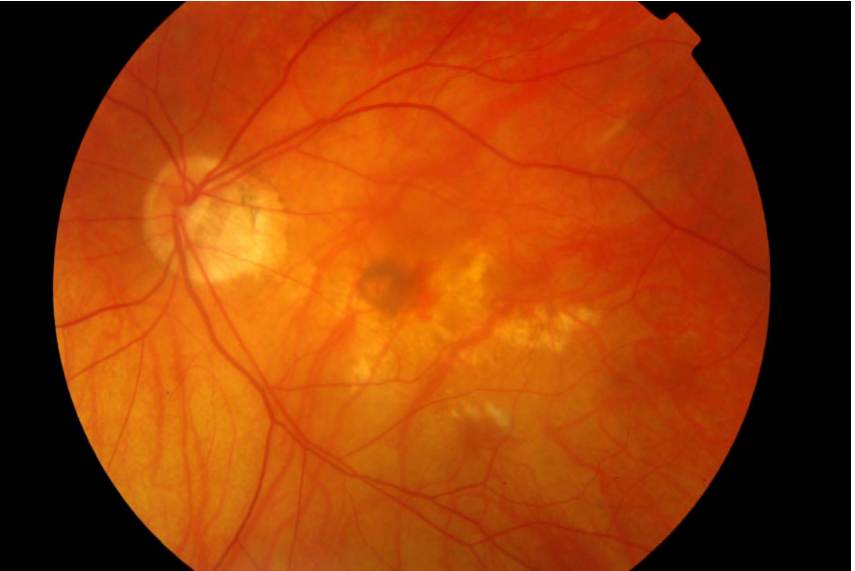
**Colour fundus photo of the left eye with myopic macular degeneration, atrophy and an elevated greyish lesion with associated pre-retinal haemorrhage**.

**Figure 2:**
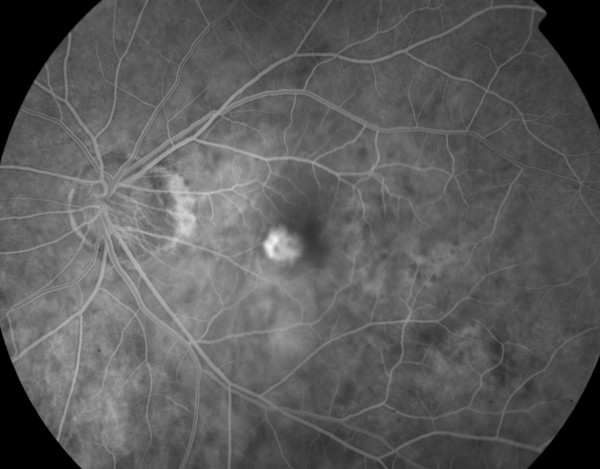
**Early phase fundus fluoroscein angiogram showing choroidal neovascular membrane with well defined hyperfluorescence**.

Examination of the left fundus revealed a myopic, tilted disc and staphyloma and an elevated grayish lesion associated with small pre-retinal haemorrhage. The vitreous was quiet and retinal vessels were of normal calibre (Figure 1). Ocular coherence tomography (OCT) showed no sub-retinal fluid but did reveal a choriodal neovascular membrane (CNVM). Fundus fluoroscein angiogram (FFA) showed a classic subfoveal CNVM, with early, well-defined hyperfluorescence (Figure 2) and late leakage. Therefore, the drop in the vision of her left eye was attributed to the development of a CNVM. Myopic changes were seen in the right eye but it was otherwise unremarkable.

**Figure 3:**
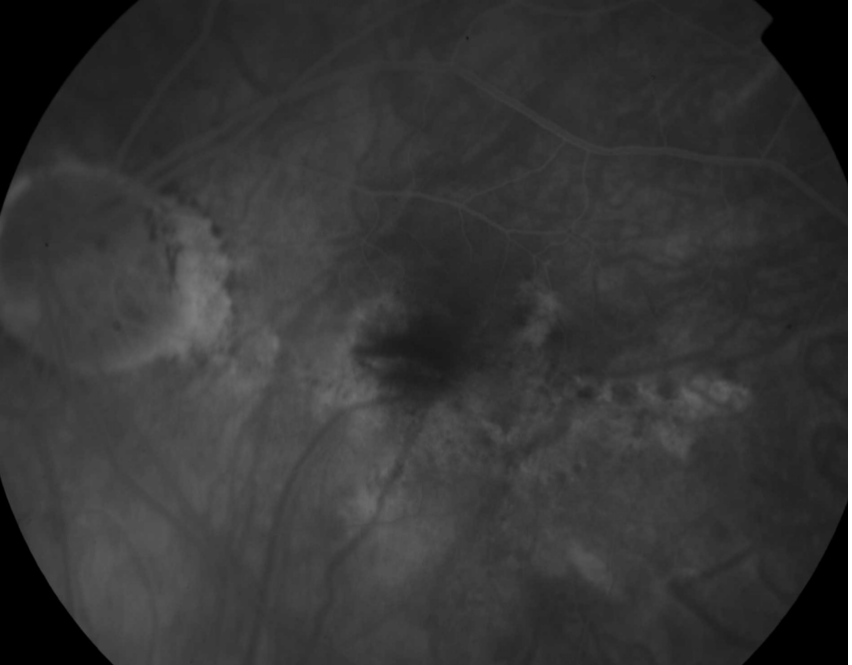
**Fundus fluoroscein angiogram showing regression of the choroidal neovascular membrane complex**.

After considering discussion of various treatment options, the patient decided to proceed with 0.5 mg of intravitreal ranizumab (Lucentis). One month following intravitreal injection into the left eye, her visual acuity improved from 6/32 to 6/12. OCT and FFA showed no subretinal fluid and furthermore regression of CNVM complex (Figure 3). However, the patient still complained of distortion and that the images are still smaller and darker in the left eye compared to the right eye.

Three months post-treatment, her visual acuity in the left eye was 6/18, improving to 6/12 with pinhole. She noticed further improvement in left reading vision. The patient re-presented four and a half months post-treatment with new distortion in the left eye. FFA showed a small area of new leakage away from the centre of vision. It was decided to withhold further treatment at that time as her visual acuity was 6/12, improving to 6/9 with pinhole, which remained stable for 16 months. The patient agreed to monitor for any changes with an Amsler chart. VA and OCT findings in her subsequent follow up appointments were stable up to one year after treatment with Lucentis.

## Discussion

Choroidal neovascular membrane is one of the leading causes of severe visual loss. Usually a manifestation in the elderly, it is often associated with age-related macular degeneration. In this case, however, it is as a cause of the myopia of the patient.

It appears the balance between antiangiogenic factors (e.g., pigment epithelium derived factor) and angiogenic factors (e.g. vascular endothelial growth factor or VEGF) determines the growth of CNV and VEGF has been temporally and spatially correlated with the development of CNV [8].

The main treatment options for CNV are photodynamic therapy, surgery and anti-vascular endothelial growth factor (anti-VEGF) treatment.

VEGF was isolated in 1989 [9] and VEGF-A is now known to promote growth of vascular endothelial cells from arteries, veins and lymphatics and is needed as a survival factor for vascular endothelial cells [10]. Eventually, in 2005 VEGF-A, a known mediator of tumour angiogenesis, was documented to have a key role in the development of the choroid vasculature. Examples of VEGF inhibitors include pegaptanib (Macugen), ranibizumab (Lucentis) and bevacizumab (Avastin).

The use in myopic CNVM of intravitreal bevacizumab (Avastin), a cheaper and closely related alternative to ranibizumab, has been reported in both retrospective [11,12] and prospective studies [13,14], with the majority of patients achieving CNVM remission and improvement in visual acuity. Currently, bevacizumab is the mainstay of management both as a mono-therapy and as an adjuvant to PDT. Furthermore, although bevacizumab appears to be a safe and effective treatment for myopic CNVM, follow-up periods have been relatively short, ranging from 35 days to seven months and long-term outcome is unknown.

In 2008, Silva *et al. *conducted a retrospective, non-randomized interventional case series study on the short term efficacy and safety of intravitreal ranibizumab for myopic CNV. A significant mean improvement in VA was noted at one, three and six months, with a significant reduction in mean central retinal thickness, as seen on OCT [15]. In addition, in 2009 a prospective study of 31 newly diagnosed patients showed a similar improvement in VA, in non-AMD related CNV with a mean follow up of 13.4 months [16]. Treatment of myopic CNVM with intravitreal ranibizumab with a 16-month-follow-up has not previously been reported in the literature. The prohibitive cost of ranibizumab has led to widespread use of bevacizumab.

Our patient had treatment with ranibizumab (Lucentis). Ranibizumab was developed due to questions over the ability of intravitreally injected molecules to penetrate across the retinal layers and reach the choroid.

The safety and efficacy of ranibizumab in the treatment of neovascular AMD have been evaluated in two large phase III, multicenter, randomized, double-masked, controlled pivotal trials, including different neovascular AMD patient populations.

The MARINA trial randomized 716 subjects in the United States with CNV to one of three treatment arms: monthly placebo injections, monthly intravitreal injections of 0.3 mg of ranibizumab, or monthly intravitreal injections of 0.5 mg of ranibizumab.

The ANCHOR trial randomized 423 subjects in the United States, Europe, and Australia who had CNV to one of three treatment arms: verteporfin photodynamic therapy with monthly placebo ocular injections, monthly intravitreal injections of 0.3 mg of ranibizumab with a placebo photodynamic therapy procedure, and monthly intravitreal injections of 0.5 mg of ranibizumab with a placebo photodynamic therapy procedure.

Analyses of these two phase III studies (ANCHOR and MARINA trials) indicate that ranibizumab results not only in a slowing down of vision loss but also a clinically meaningful vision gain at the primary 12-month assessment in a significant proportion of patients. In the case of the MARINA study these benefits were also observed through the final 24-month assessment [17].

In 2007 an open-label single centre prospective study called the prospective OCT imaging of patients with neo-vascular AMD Treated with intra-ocular Lucentis (PrONTO) was designed to investigate the role of OCT in guiding retreatment decisions for a variable dosing regimen in patients with choroidal neovascularisation (CNV) secondary to AMD. The aim of the study was to find out if an OCT-guided treatment regimen could be used to maintain improvements in visual acuity over two years after three consecutive monthly doses of Lucentis (500 μg) [18].

The results showed rapid improvements in visual acuity and OCT measurements. After 12 and 24 months, outcomes in the study were similar to the MARINA and ANCHOR phase III study results. It is worth noting that the mean frequency of dosing reduced by more than half. Based on these results, OCT appears to be a useful tool for guiding retreatment decisions such as the frequency of treatment of patients with CNV. However a prospective, randomized clinical trial is needed to confirm these results [18].

## Conclusion

In conclusion, we presented a patient with myopic CNVM whose vision improved and stabilized at 6/6 after one treatment of ranibizumab (Lucentis). Furthermore, a review of major trials that have been done on CNV show that ranibizumab in CNV not only reduces loss of vision but in fact results in visual gain. In addition, the PrONTO trial shows that the frequency of treatment should be guided by investigations such as OCT and the treatment tailored to the individual findings in the patient (such as an increase in central retinal thickness of 100 μm or more on OCT.) This is further supported by studies showing the use of intravitreous anti-VEGF resulting in long term remission of other Type 2 CNVM.

Due to the relative rarity of myopic CNVM, there is lack of evidence for intravitreal anti-VEGF treatment. Treatment of CNVM should therefore be individualized and the chance of spontaneous resolution discussed with patients. This case report presents intravitreal ranibizumab as a reasonable treatment option, and shows that the frequency of treatment can be modulated according to OCT findings.

## Abbreviations

CNV: choroidal neovascularisation; CNVM: choroidal neovascular membrane; OCT ocular coherence tomography; FFA: fundus fluoroscein angiogram; VEGF: vascular endothelial growth factor.

## Competing interests

The authors declare that they have no competing interests.

## Authors' contributions

DS reviewed the patient in clinic. AT and DS structured the management plan and followed up the patient. NK and DS reviewed the article for intellectual content while NK carried out a literature review. NK and DS read and approved the final script.

## Consent

Written informed consent was obtained from the patient for publication of this case report and any accompanying images. A copy of the written consent is available for review by the journal's Editor-in-Chief.
